# Shared effects of the opioid antagonist naltrexone on first-hand and empathic pain

**DOI:** 10.1093/scan/nsag018

**Published:** 2026-03-23

**Authors:** Julia T Braunstein, Markus Rütgen, Claus Lamm

**Affiliations:** Department of Cognition, Emotion, and Methods in Psychology, Faculty of Psychology, University of Vienna, 1010 Vienna, Austria; Vienna Cognitive Science Hub, University of Vienna, 1010 Vienna, Austria; Department of Cognition, Emotion, and Methods in Psychology, Faculty of Psychology, University of Vienna, 1010 Vienna, Austria; Vienna Cognitive Science Hub, University of Vienna, 1010 Vienna, Austria; Department of Cognition, Emotion, and Methods in Psychology, Faculty of Psychology, University of Vienna, 1010 Vienna, Austria; Vienna Cognitive Science Hub, University of Vienna, 1010 Vienna, Austria

**Keywords:** empathy, shared representations, psychopharmacology, naltrexone, pain

## Abstract

Empathy allows us to infer the affective state of another individual and resonate with it. Accumulating evidence suggests that on a neural level, empathizing relies on “shared neural representations”, i.e. patterns of neural activation recruited both during the first-hand and the empathic experience of a specific affective state. Studies employing placebo analgesia have shown consistent reductions in behavioral ratings and neural activity for both firsthand and empathic pain. The mechanistic interpretation of such effects, however, remains elusive, as placebo analgesia could exert its effects on empathy either via pharmacological actions or via top-down cognitive processes on pain and empathy beliefs. To address this limitation, this double-blind placebo-controlled study (*N* = 35, 21 females) administered the opioid antagonist naltrexone and tested its effects on firsthand pain and affective and cognitive ratings of empathy for pain. While we predicted that naltrexone would increase both electrocutaneous and cold pain, as well as cognitive and affective aspects of empathy for pain, the results instead pointed in the other direction. While these hypo- rather than hyperalgesic effects were unexpected, the coherence in their directionality fits with previous findings and suggests the involvement of shared opioidergic mechanisms in the firsthand experience of pain and empathy for pain.

## Introduction

Empathy plays a key role in social interaction. It allows one to infer the affective state of another individual and vicariously share their experience (de Vignemont and Singer 2006). A prominent theory in social neuroscience suggests that the ability to empathize is based on an overlap of neural and bodily sensations experienced during both firsthand and empathic affective experiences, indicating a similar neural mechanism to underpin both self- and other-related experiences (Marsh 2018; Preston and de Waal 2002; Fallon *et al*. 2020). This notion is most commonly referred to as the shared representations account, with recent theorizing suggesting shared representations on different levels of granularity, from generalized affective arousal to affect valence to domain-specific features (Rütgen and Lamm 2024).

Neuroimaging and electroencephalography studies consistently demonstrated evidence for shared representations via partially similar brain responses during both firsthand and empathic pain (e.g. (Lamm *et al*. 2011; Riečanský and Lamm 2019). A central limitation of these methods is that they are correlational in nature, implying that we cannot infer that the same neural mechanisms are at play from the mere similarity in brain activations or their electrophysiological signatures. Causal psychopharmacological approaches have thus been introduced to test if experimentally modulating the firsthand experience of pain will also impact pain empathy, demonstrating that participants showing placebo analgesia also showed reduced pain empathy (Rütgen *et al*. 2015a, 2015b; Rütgen *et al*. 2018). In addition, administering the opioid antagonist naltrexone abolished these effects, leading to the conclusion that the body’s endogenous opioid system is implicated not only in the regulation of firsthand pain but also in empathy for someone else’s pain (Rütgen *et al*. 2015b). While these studies are all from our laboratory, awaiting independent replication by other laboratories, similar findings have been reported when administering the painkiller acetaminophen (Mischkowski *et al*. 2016).

Although these studies support the notion of shared representations, the use of placebo analgesia, and to a certain extent also of acetaminophen, cannot address one major challenge: should the findings be explained by pain-specific “neuropharmacological” mechanisms, or by top-down “psychological” effects (Atlas 2021), such as treatment expectations or other types of beliefs about how the placebo or real painkiller will affect pain perception and the related subjective experiences. Such expectations play a prominent role in pain, with some studies showing that positive expectations may account for up to 50% of the analgesic effects of the medication used in clinical trials (Amanzio *et al*. 2001; Bingel *et al*. 2011; Schmidt *et al*. 2022).

To address this limitation, we conducted a double-blind placebo-controlled psychopharmacological study in which we administered the opioid antagonist naltrexone, using a within-subject crossover design. By administering an opioid antagonist, we intended to block the receptor-binding of endogenous opioids, which are released upon nociception (Corder *et al*. 2018; Bagley and Ingram 2020), and thereby increase pain sensitivity in our participants (e.g. Omara-Reda *et al*. 2022). With this approach, we aimed to test more specifically whether directly manipulating opioidergic pain regulation will show similarly coherent effects on both firsthand pain and empathy for pain. A central aspect of our design was to administer the substance without informing participants about their name, pharmacological effect, and content. This aimed to minimize potential effects of any (placebo- or instruction-based) beliefs and thus reduced expectations about whether and how the substance would impact pain, affect, or cognition. We hypothesized that naltrexone (vs. placebo) would increase firsthand pain, and, correspondingly, also increase empathy for pain.

## Materials and methods

### Participants

This study was conducted in accordance with the latest revision of the Declaration of Helsinki (World Medical Association, 2013) . To investigate empathy for another individual’s pain and limit potential expectancy effects related to the administered substances, participant deception was necessary and approved by the local ethics committee (EK 661/2011). Before providing written consent, participants received all relevant study information (except for the elements of deception). At the end of the study, participants were fully debriefed, and they received 90€ as compensation for their time.

After a medical screening, eligible participants were invited to the study sessions (see Supplementary for full list of criteria). While 42 healthy right-handed volunteers were included, only the data of 35 (21 females; mean ± SEM = 23.46 ± 2.82 years) could be analyzed due to malfunctions of the electrical pain stimulator during testing of seven participants. A sensitivity analysis conducted in G*Power 3.1 (Faul *et al*. 2007), based on the final sample size and the planned dependent samples *t*-tests, indicated that the minimum detectable effect size with 80% power was *d* = 0.43.

### Study design and procedure

The present study was designed as a double-blind within-subjects crossover experiment. Participants completed the same experimental procedure twice, the only difference being the administered substance. While in one session participants received naltrexone (50 mg Dependex^®^), in the other they were administered a placebo (maltodextrin). Participants were informed that the pills they were given served as contrast agents intended to enhance the fMRI signal, without disclosing their true nature. The assignment of either substance to the experimental sessions was randomized by a colleague not involved in this study, with drug order (naltrexon-placebo; placebo-naltrexone) counterbalanced. Due to the exclusion of some participants, this order was slightly disbalanced in the end (*N*_naltrexone-placebo/placebo-naltrexone_ = 16/19).

Both experimental sessions took place at least 1 week apart. Each session started with the participant and a confederate of the researchers arriving at the laboratory. After a short introduction, they were escorted to two different rooms to (allegedly) undergo some preparatory measures.

The experiment then started by calibrating a participant’s individual pain thresholds, following a frequently used calibration protocol (Rütgen *et al*. 2015a; Hartmann *et al*. 2022), to establish which stimuli participants considered “barely noticable” (no-pain) and “very painful, but bearable” (pain). Next, participants were administered the drug (or placebo), followed by a waiting period of 45 minutes. The waiting period followed governmental recommendations for Dependex^®^ and NIH guidelines for ReVia^®^/Depade^®^, which indicate peak plasma levels at ∼60 minutes. Because we aimed for the peak to coincide with the empathy for pain paradigm, the cold pressor task (CPT) was performed earlier, after 45 minutes post-intake, when plasma levels were expected to be high but not yet maximal. Then, participants completed the CPT, after which they were placed inside the scanner and then completed the empathy for pain paradigm, followed by another unrelated task whose findings are presented elsewhere (Zhao *et al*. 2021). At the end of session two, belief in the study setup was checked via an open interview, and only participants reporting no doubts remained in the sample. Probably due to a persistent technical issue with scanner synchronization that was only noticed after all data had been collected, fMRI analyses of the empathy for pain paradigm did not yield meaningful activations, but the behavioral data were not affected by this issue and are thus focused on here.

### Experimental paradigms

#### Cold pressor task

The CPT was included to test whether administration of naltrexone would lead to greater pain sensitivity as compared to placebo. Participants were instructed to submerge their right hand in cold water and report from when their hand started to hurt. Experimenters recorded (in seconds) how long it took for participants to experience pain (*CPT threshold*), as well as how long they could keep their hands submerged in the water (*CPT tolerance*). For a more detailed description of the protocol, see Supplementary section 2.4.

#### Empathy for pain paradigm

**Figure 1:**
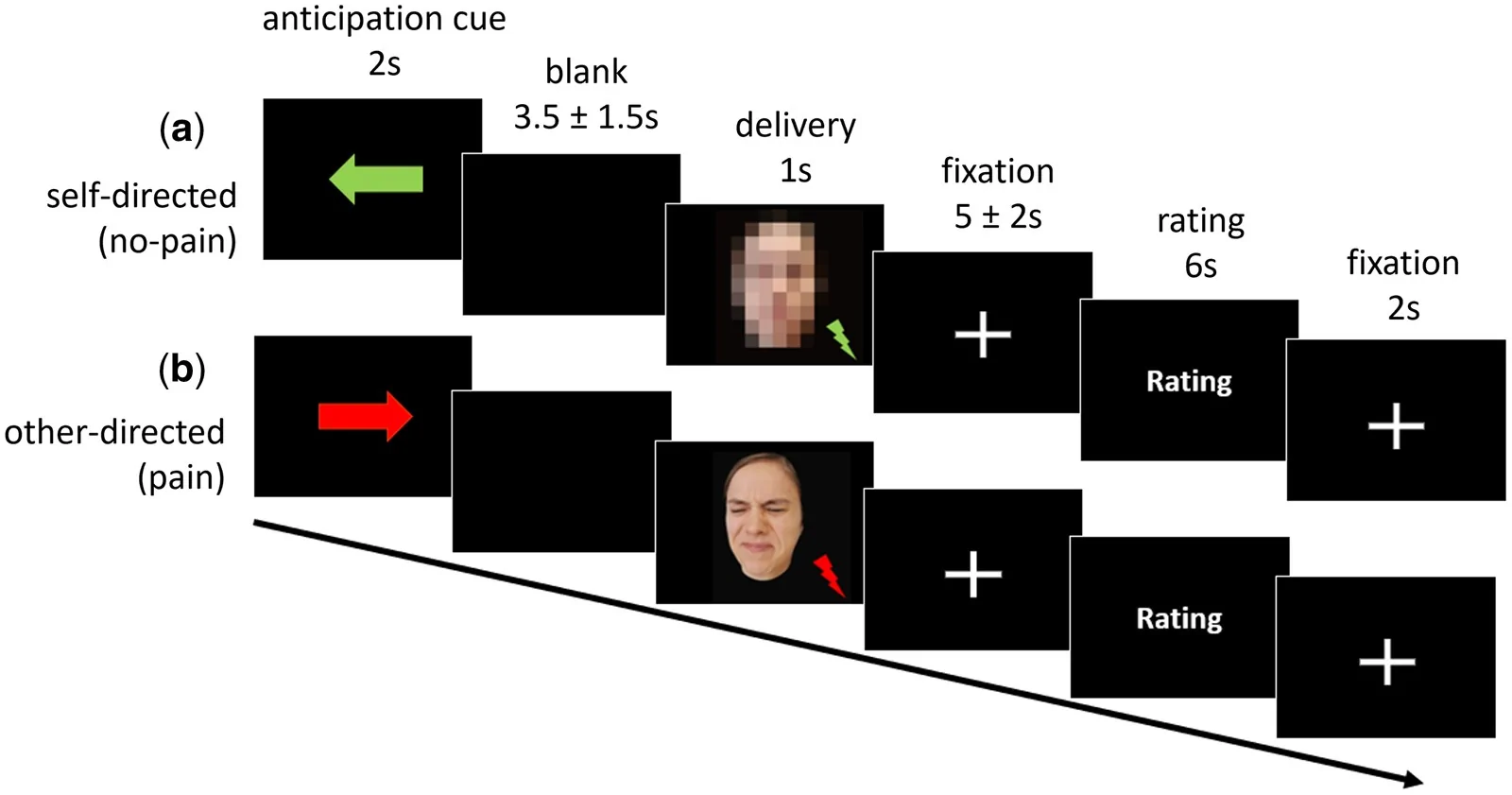
Structure and timeline of two example trials. (a) self-directed no-pain trial, (b) other-directed pain trial. First the anticipation cue indicated the recipient (pointing left = self, pointing right = other), as well as the intensity of the next stimulus (green = non-painful, red = painful). Then a black screen was shown for a jittered duration (3500 ± 1500 ms). Next, the electrical stimulus was delivered (500 ms) while a picture was presented (1000 ms). The picture showed either a scrambled face (self-directed trials) or the confederate with a relaxed or painful facial expression (other-directed trials), along with a small lightning symbol in red (painful stimulus) or green (non-painful stimulus). In a third of the trials, ratings (6000 ms per rating) were collected.

An empathy for pain paradigm from our laboratory (e.g. Rütgen *et al*. 2018; Wagner *et al*. 2020; Rütgen *et al*. 2021), adapted from Singer *et al*. (2004), was employed to assess the main hypotheses of this study. In prior work, this paradigm reliably elicited similar brain responses for firsthand and empathic pain alongside behavioral measures sensitive to opioidergic modulation. Thus, this paradigm’s established procedures and a strong backbone of prior findings seemed ideally suited to be used in the present study. Participants either underwent firsthand painful or non-painful electrical stimulation or witnessed a confederate (allegedly) receive such stimulation. At trial outset an anticipation cue (duration = 2000 ms) indicated the recipient (self vs. other) and the intensity (pain vs. no-pain) of an upcoming stimulus. Following a short jitter period (3500 ms ± 1500 ms; blank screen) the electrical stimulus was delivered for 500 ms. Visual stimuli were presented in parallel for 1000 ms. When the confederate was the recipient of the stimulus, participants were shown a picture of the confederate’s face, with the facial expression varying depending on stimulus intensity (relaxed or painful). If participants themselves received the stimulus, they were presented with a scrambled face picture to control for visual stimulation. Moreover, a small lightning symbol in the bottom right corner of the screen served as an additional reminder of stimulus intensity. Next, another jitter period (5000 ms ± 2000 ms) followed wherein a fixation cross was presented. In a third of the trials, participants were asked to provide ratings on a seven-point Likert scale (6000 ms rating time per question). Fifty percent would concern their own pain, asking “How painful was this stimulus for you?”, with the scale ranging from “not at all” to “extremely painful” (*self-directed pain* ratings). During the other 50%, participants were either rating the other persons’ pain, asking “How painful was this stimulus for the other person?”, ranging again from “not at all” to “extremely painful” (henceforth labeled as *other-directed pain* ratings), or their own affect in response to witnessing the other persons’ stimulation, asking “How unpleasant did it feel when the other person was stimulated?” on a scale ranging from “not at all” to “extremely unpleasant” (*empathic unpleasantness* ratings). Before the start of the next trial, a fixation cross was presented (2000 ms). In sum, 60 trials with 15 trials per condition (self-directed pain/no pain; other-directed pain/no pain) and 5 ratings per condition were used. Trial conditions and ratings were ordered in a pseudo-randomized manner, with a maximum of three repetitions of each condition (Fig. 1).

The rationale for this task setup in prior studies and the present work was to investigate responses to firsthand pain and empathy for pain in a closely matched setting. Administering painful electrical stimuli not only to the other, physically present and previously encountered person, as well as to the participants themselves allows a direct connection of firsthand pain to pain empathy experiences, as captured by intensity and unpleasantness ratings. The latter were used to assess two distinct aspects of empathy, the cognitive-evaluative aspect and the affective-motivational aspect. While the former consists of an other-related rating capturing the intensity of pain attributed to the other person, the latter is a self-related rating capturing a participant’s own affective response while witnessing the other person being in pain.

### Data analysis

Data analysis was conducted using RStudio (R version 4.4.1). As we expected an increase in pain sensitivity following the administration of naltrexone as opposed to a placebo, we expected a decrease in both CPT threshold and tolerance. To test this hypothesis, we employed two Wilcoxon signed-rank tests (one-tailed), comparing mean CPT threshold and tolerance across the two sessions (naltrexone vs. placebo). For the empathy paradigm, we expected participants to report higher ratings for painful stimuli administered to themselves (self-directed pain) and to the other person (other-directed pain), as well as greater unpleasantness when witnessing painful stimuli administered to the other person (empathic unpleasantness). To test these hypotheses, we conducted two repeated measures (rm) ANOVAs to assess the overall pattern of findings (one for the pain ratings, the other for the empathic unpleasantness ratings) as well as planned comparisons (dependent samples *t*-tests comparing pain ratings during naltrexone and placebo, calculated using the differences between pain and no-pain for self-directed pain, other-directed pain, and empathic unpleasantness ratings). In the pain ratings rmANOVA, within-subject factors included drug (naltrexone vs. placebo), pain intensity (pain vs. no-pain), and recipient (self vs. other), with the self-related pain and other-related pain ratings as dependent variables; for the unpleasantness ratings rmANOVA, within-subject factors were the same, and empathic unpleasantness ratings was the dependent variable. Due to violation of the sphericity assumption all rmANOVA results are reported using Greenhouse-Geisser sphericity correction. We set the significance threshold at *P *< 0.05 and additionally report two-sided *P*-values for planned contrasts that yielded significant effects. Since the effect sizes reported in the results section were generally below our minimum detectable threshold (*d* = 0.43), we complemented each frequentist analysis with its Bayesian equivalent. Detailed results of the Bayesian analyses are provided in the respective sections of the supplement. Additionally, we explored whether the effects of drug (naltrexone vs. placebo) on either of our empathy measures (other-directed pain ratings and empathic unpleasantness ratings) would be mediated by changes in firsthand pain experienced by participants (self-directed pain ratings).

Note that multiple-comparison correction was applied only for exploratory post hoc comparisons. For analyses testing a priori hypotheses targeting distinct constructs—such as effects on affective versus cognitive dimensions of empathy—we interpret each result independently rather than as part of a single family of tests; therefore, family-wise error control was not performed for such analyses (Rubin 2021; Barnett *et al*. 2022).

## Results

### No effect of naltrexone on CPT threshold or tolerance

**Figure 2:**
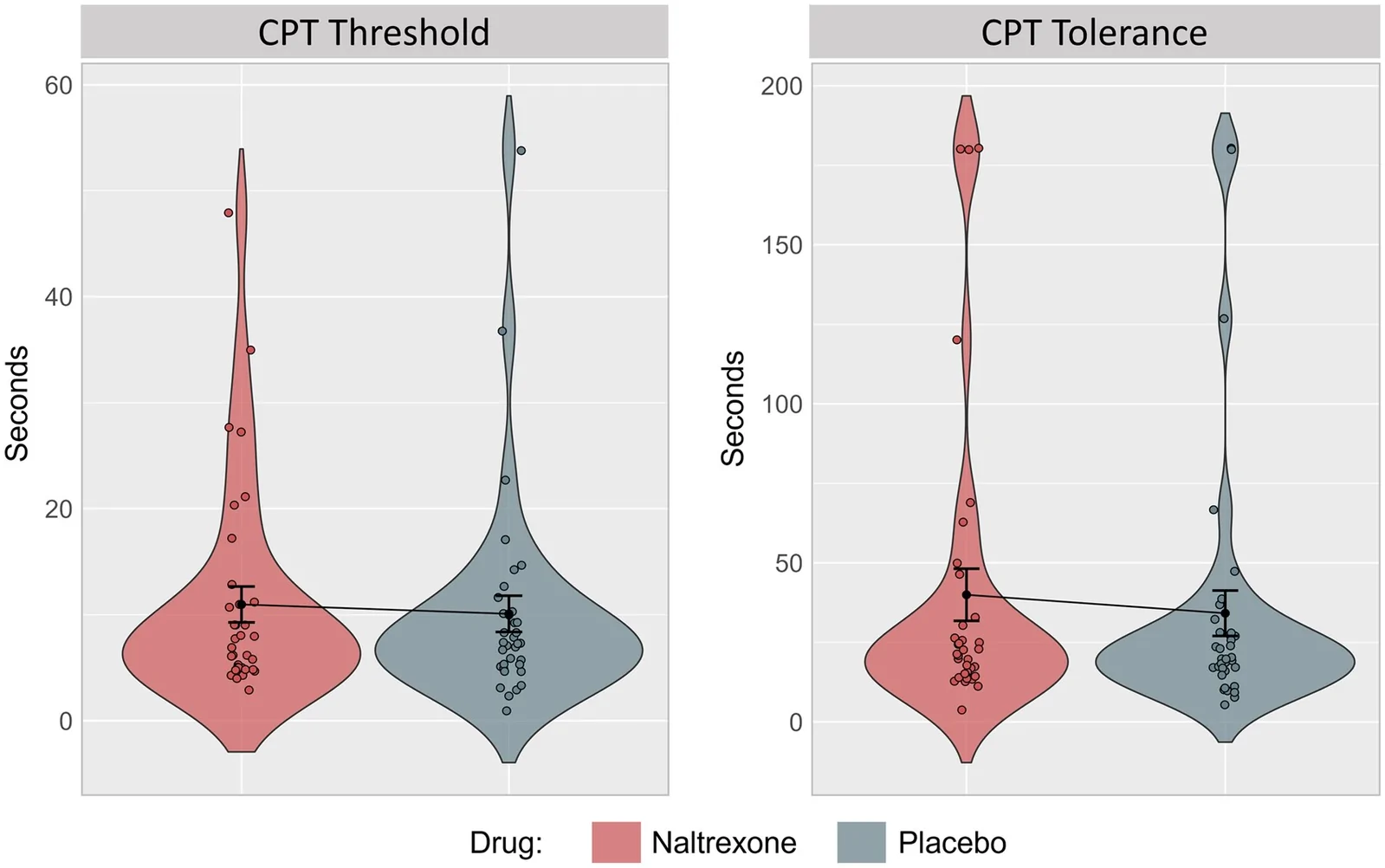
Cold pressor task (CPT) data (*n* = 35). Left: comparison of the CPT threshold (how long it took until participants experienced pain while having their hand submerged in cold water, measured in seconds) between the two drugs. Right: comparison in CPT tolerance (how long participants were able to keep their hands submerged in the cold water before they had to remove it, measured in seconds) between the two drugs.

Wilcoxon signed-rank tests comparing mean CPT threshold and tolerance between the two drugs revealed no significant difference [CPT threshold: *W* = 234.50, *P *= .293, *r* = -0.11, 95% CI (-1.00, 0.20); CPT tolerance: *W* = 197.50, *P *= .136, *r* = -0.20, 95% CI (-1.00, 0.11)] (Fig. 2).

### Naltrexone decreases firsthand pain and empathic unpleasantness, but not other-directed pain ratings

**Figure 3:**
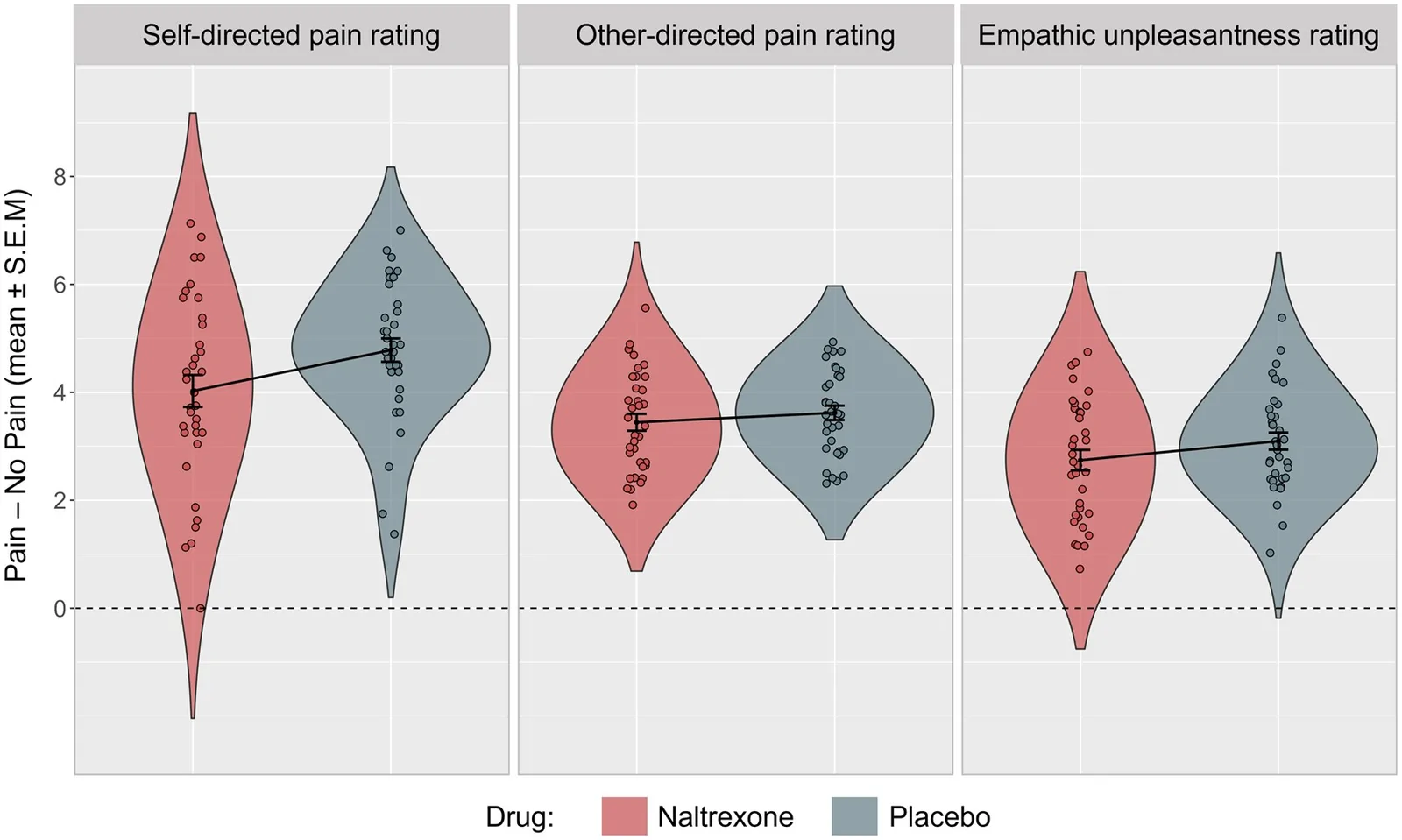
Self-report results of the empathy for pain task, for ratings of self-directed pain (“How painful was this stimulus for you?”), other-directed pain (“How painful was this stimulus for the other person?”; cognitive-evaluative aspect of empathy), and empathic unpleasantness ratings, of negative affect participants themselves experienced when witnessing the other person’s pain (“How unpleasant did it feel, when the other person was stimulated?”; affective-motivational aspect of empathy).

The rmANOVA conducted on the cognitive-evaluative aspects of empathy, captured by other-related pain ratings, revealed a significant main effect of intensity [*F*(1,34) = 699.516, *P *< .001, *η_p_^2^* = 0.954], indicating that painful stimuli were rated as significantly more intense, as compared to non-painful stimuli. Additionally, an interaction effect was found between drug and intensity [*F*(1,34) = 7.444, *P *= .01, *η_p_^2^* = 0.180], with post-hoc comparisons showing a selective reduction for painful stimuli under naltrexone versus placebo. A significant interaction between recipient and intensity [*F*(1,34) = 24.116, *P *< 0.001, *η_p_^2^* = 0.415] revealed a higher difference between ratings related to painful stimulation experienced firsthand versus by the other person, compared to differences in ratings related to non-painful stimulation (see Supplementary section 2.5 for post-hoc comparisons). There was no significant main effect of drug and no interaction of drug with either recipient or intensity. The three-way interaction between drug, recipient, and intensity also was not significant [*F*(1,34) = 3.328, *P *= .077, *η_p_^2^* = 0.089]. Planned comparisons for the difference in pain ratings showed a significant decrease in ratings of self-directed pain in the naltrexone session, as compared to the placebo session [self-directed: paired *t*-test, *t*(34) = -2.54, one-tailed *P *= .008, two-tailed *P *= .016, Cohen’s *d* = -0.43], while the planned comparison of the cognitive-evaluative aspects, as captured by *other-directed pain ratings* (pain minus no-pain; naltrexone vs. placebo), revealed no significant effect [paired *t*-test, *t*(34) = -1.23, *P *= .113, one-tailed, Cohen’s *d* = -0.21] (Fig. 3).

The rmANOVA on the affective-motivational aspect of empathy, captured by the empathic unpleasantness ratings, revealed a main effect of intensity [*F*(1,34) = 363.206, *P *< 0.001, *η_p_^2^* = 0.91440]. Moreover, there was a significant interaction between drug and intensity [*F*(1,34) = 4.432, *P *= 0.043, *η_p_^2^* = 0.115]; however, post-hoc comparisons indicated that none of the pairwise comparisons showed a statistically significant difference (see Supplementary section 2.5). Planned comparisons confirmed a significant decrease in the affective-motivational aspect [empathic unpleasantness: paired *t*-test, *t*(34) = -2.11, one-tailed *P *= .021, two-tailed *P *= .043, Cohen’s *d* = -0.36] (Fig. 3). This suggests that under naltrexone, participants experienced less negative affect when seeing the other person in pain, implying a reduction in affective-motivational aspects related to empathy.

### Naltrexone effects on empathy measures are mediated by changes in firsthand pain

Mediation analyses were conducted using the mediation package in R (Tingley *et al*. 2009) with 1000 bootstrap iterations. The first model (m1) tested whether drug (naltrexone vs. placebo) affected cognitive-evaluative aspects of empathy (other-directed pain ratings) via changes in firsthand pain (self-directed pain ratings); the second model (m2) tested affective-motivational aspects of empathy (empathic unpleasantness). In both models, the indirect effect was significant (m1: b’ = 0.176, *P *= .032; m2: b’ = 0.206, *P *= .024) while the direct effect was not (m1: c’ = –0.0003, *P *= .996; m2: c’ = 0.148, *P *= .534), indicating that changes in firsthand pain mediated the effects of naltrexone on empathy (see Supplementary section 3.5).

## Discussion

This study aimed to investigate a potential shared opioidergic mechanism between the firsthand and empathic experience of pain. To this end, we manipulated endogenous pain modulation mechanisms via administration of the opioid antagonist naltrexone, while also minimizing drug expectations by hiding the drug name and effect. We hypothesized that blocking opioid receptors and thereby impeding the binding of endorphins for pain relief would result in heightened pain sensitivity, compared to the placebo session. In line with shared representations accounts of empathy, we assumed corresponding effects on pain empathy. Our findings indicate that contrary to inducing hyperalgesia, naltrexone had the opposite effect: participants rated their own pain as less intense compared to the placebo session. As regards empathy, results are mixed: while the cognitive-evaluative other-directed pain ratings were not significantly affected, the ratings of empathic unpleasantness, which capture affective-motivational aspects of empathy, showed a similar decrease as the self-pain rating. Moreover, an explorative mediation analysis revealed no direct effect of naltrexone on either component of empathy, but significant indirect effects emerged when changes in self-directed pain were considered as a mediator. Due to the absence of a direct effect, the mediation results should be interpreted with caution; nevertheless, the overall pattern of our findings lends partial support to the hypothesis of a correspondence of self-pain and empathic pain processing.

These findings support the notion that the ability to empathize with another individual’s affective state is partially grounded in the experience of that state in oneself. That said, we only partially reproduced the findings of prior studies using the same paradigm. In prior work, induction of placebo analgesia not only affected participants’ firsthand experience of pain from electrical stimuli, but also their perception of how painful such stimuli were for another person, i.e. the cognitive-evaluative aspect of empathy captured by the other-directed pain ratings. Additionally, placebo analgesia affected ratings of unpleasantness while witnessing another person in pain, indicative of changes in affective-motivational responses to the pain of others as well. Notably, these effects were significantly reduced or “blocked” when naltrexone was administered after induction of placebo analgesia (see references Rütgen *et al*. 2015a, 2015b).

In the present study, administration of naltrexone only significantly affected firsthand pain and the affective-motivational dimension of empathy, but not its cognitive-evaluative aspect. This may be due to our specific study aim. We employed a double-blind design with extant deception regarding the content and effects of the administered pharmaceutical, which was conveyed to participants as an “MRI signal amplifier.” With this approach, we aimed to focus exclusively on the “pure” neuropharmacological mechanisms underlying firsthand pain regulation and its relation to empathic pain. The associated reduction of beliefs and expectations regarding the administered substance or the intention of the study may explain the differences observed between the present study and previous research from our laboratory. In the preceding studies, placebo analgesia was induced by creating the belief that participants received a pill with strong analgesic effects. This led to pain relief expectations, which modulate the experience of pain through top-down regulatory mechanisms (Peerdeman *et al*. 2016; Ortega *et al*. 2022). Moreover, participants’ beliefs that they had received pain medication was incongruent with the information given about the other person (“the other person did not receive any medication”). This mismatch may have influenced experimental demand effects related to the transfer of pain relief, potentially leading to egocentrically biased judgments when rating the other person’s pain (Epley *et al*. 2004; Hoffmann *et al*. 2016). In contrast to our previous studies, the double-blind pharmacological design in the present study aimed to minimize the formation and adjustment of pain-related beliefs and expectations.

Of note, other studies that employed acute blinded administration of various substances, such as psilocybin (Pokorny *et al*. 2017; Mason *et al*. 2019), LSD (Dolder *et al*. 2016; Holze *et al*. 2021), MDMA (Hysek *et al*. 2014; Schmid *et al*. 2014; Kuypers *et al*. 2017), and alcohol (Maurage *et al*. 2011; Francis *et al*. 2019), have consistently shown significant effects limited to affective-motivational aspects of empathy, with no corresponding effects observed in the cognitive-evaluative domain. Only one study deviated from this pattern, showing significant effects of LSD on both aspects of empathy (Dolder *et al*. 2016). Similarly, previous work has shown that naltrexone reduces negative affective responses to others’ pain (Haaker *et al*. 2017) as well as sensitivity to others’ negative emotional expressions (Wardle *et al*. 2016), further suggesting that its effects are most pronounced on the affective dimensions of empathy. Without conflicting expectations and cognitive biases, participants have to rely on the recruitment of representations on a level that is more specific to the state in question to make their judgments about someone else (Rütgen and Lamm 2024). This implies that in a setting with minimized treatment expectations, as was the case in our double-blind pharmacological design, specifically only the level of shared opioidergic pain regulation was modulated, to make judgments about the pain of others. This aligns with the concept of shared representations discussed in our recent review (Rütgen and Lamm 2024), highlighting that pain empathy operates on multiple levels. Furthermore, our findings indicate that modulating the pain-specific level through naltrexone is sufficient to exert effects on both firsthand pain and the affective-motivational processing of another person’s pain. Comparing the prior findings using placebo analgesia as well as other substances with the fact that other-pain ratings were not, but empathic unpleasantness ratings were influenced by naltrexone, highlights two important aspects: On a conceptual level, it seems that naltrexone and the opioidergic blockade it entails acts mainly on the affective component of empathic pain processing. That the affective component of empathy is more sensitive to being manipulated is in line with previous neuroimaging and EEG results of our lab, which predominantly showed effects on affective-motivational neural networks and ERP signatures (Rütgen *et al*. 2015a, 2018). We thus speculate that naltrexone predominantly affected these networks, translating into selective effects only on the affect-related empathic unpleasantness ratings. Of note, we did not ask participants to also rate their firsthand pain unpleasantness, but only their intensity, which would have allowed to test this hypothesis more specifically. However, previous work has shown that firsthand pain intensity and unpleasantness are highly correlated, and that participants often incorporate pain unpleasantness rather than “pure” intensity in their pain intensity ratings, which is why we abstained from asking for separate rating (Price 2000, 2002). Second, the selective effects on only one type of rating allows us to exclude a possible confound: if naltrexone would have led to an overall decrease or dampening of any kind of subjective experience and the associated ratings of that experience, for instance because of sedative side-effects or cognitive impairments, it should also have impacted on the other-directed pain ratings, which was however not the case. This strengthens our conclusion of a selective effect on affective-motivational processing.

While we initially hypothesized that administering naltrexone would lead to an increase in pain sensitivity, the results of the present study do not support this assumption. Although the opioid antagonist did not affect participant’s pain sensitivity in the Cold Pressor Task, we did observe a significant effect in their subjective ratings regarding electrocutaneous stimuli administered during the empathy paradigm. Here, participants rated their own pain as less intense under naltrexone, as compared to a placebo pill. Based on theoretical considerations, it seems reasonable to assume that blocking opioid receptors would impair endogenous pain regulation, and this was also the reason why we hypothesized hyperalgesia. We can only speculate on the reasons for the opposite finding of hypoalgesia. A closer look into the literature, for instance, reveals some inconsistencies and added insights into how naltrexone may act on pain, depending on the context within which the substance was administered (Werner *et al*. 2015). For instance, administration of naltrexone (or its intravenous counterpart naloxone) following experimental activation of the endogenous opioid system produces mostly consistent findings of reduced analgesia or pain relief, as compared to the respective control conditions (Grevert *et al*. 1983; Willer *et al*. 1990; Amanzio and Benedetti 1999; Eippert *et al*. 2009; King *et al*. 2013; Rütgen *et al*. 2015; Sirucek *et al*. 2021). However, when naltrexone is administered without prior activation of the endogenous opioid system, the results are highly variable: some studies reported hyperalgesia (Schobel *et al*. 1998; France *et al*. 2007; Schoell *et al*. 2010; Price *et al*. 2016), while others found hypoalgesia (Greeley *et al*. 1988; Foo and Westbrook 1993; France *et al*. 2007), or no effect (Cook *et al*. 2000; Ring *et al*. 2008; Younger *et al*. 2009). Currently, no decisive factors have been pinpointed to explain such heterogeneous findings. While some studies have observed a potential sex difference (al’Absi *et al*. 2004; France *et al*. 2005) and others have proposed an interaction with blood pressure (McCubbin and Bruehl 1994; Kotlyar *et al*. 2008), neither factor had an impact in our data (see Supplementary section 3 for exploratory data analyses). What remains from this closer look at the literature is that various yet to-be-precisely understood factors may influence the effects of naltrexone and opioidergic antagonism on firsthand pain. Of importance, however, the main aim of our study was to assess the correspondence of effects and to more directly assess a potentially shared opioidergic mechanism on firsthand pain and empathic pain—which our findings indeed suggest.

Our study comes with some limitations that should be considered when interpreting these results. During data collection, we encountered some technical malfunctions of the pain stimulator, leading to the exclusion of seven participants from the final analysis, which weakened the statistical power of our study. Moreover, the revealed effect sizes are small, which may be attributed to the inconsistent effects of naltrexone across studies and participants and could explain the lack of a statistically significant effect on participants’ sensitivity in the CPT. Future research should aim to systematically collect data on potential confounding factors to better understand their role in the variability of naltrexone’s effects on firsthand pain (Leknes *et al*. 2024). Additionally, the 45-minute waiting period between administration of naltrexone and the CPT may not have allowed sufficient time for adequate opioid receptor blockade to develop, potentially limiting the detection of drug effects. We recommend waiting at a minimum of 1 hour before starting any psychometric measurements in future work (see Supplementary for further elaboration). Lastly, the self-report paradigms implemented in this study do not allow for a direct observation of the drug effect. While self-report is a valid and commonly used method in substance research, it provides only limited insight into the underlying biological mechanisms. It is thus particularly unfortunate that the planned neuroimaging data of this study were not interpretable due to technical issues. The reliance on subjective ratings alone restricts our ability to draw definitive conclusions about the mechanistic role of opioidergic pathways in firsthand and empathic pain. This is noteworthy given that naltrexone blocks mu and kappa opioid receptor subtypes, each of which may contribute differently to the experience of pain and its related affective processes (e.g. Paul *et al*. 2021). Our design could not separate these mechanisms, highlighting an open question for targeted experimental work. Incorporating neurobiological measures, especially multimodality imaging approaches that combine positron emission tomography to trace opioidergic receptor activity with fMRI (Sauter *et al*. 2010) in future studies could provide a more comprehensive understanding and strengthen the interpretation of drug effects observed in self-report data.

In conclusion, our study provides evidence for a shared opioidergic mechanism underlying both firsthand pain and empathy for pain, as indicated by the consistent effects of naltrexone across both experiences. Our results further support the notion that painful experiences are shared across a variety of representational levels (Rütgen and Lamm 2024). Although the direction of the naltrexone effect, as well as its strength, were not as anticipated, the observed reduction in both pain sensitivity and empathic unpleasantness suggests a partially shared reliance of firsthand pain and empathy for pain in the affective domain, which seems plausibly linked to opioidergic mechanisms. With ultra-low-dose naltrexone increasingly emerging as a treatment option for chronic pain (Kim and Fishman 2020), it is essential to continue investigating its effects beyond pain sensitivity. Our findings suggest that some of these effects may carry over into how individuals perceive the pain of others, potentially impairing the ability of those treated with naltrexone to empathize effectively. This could have negative consequences for social relationships, emphasizing the need for further research into the broader implications of naltrexone treatment in the context of social cognition, while refining methodological approaches to better capture the nuances of such interactions.

## Supplementary Material

nsag018_Supplementary_Data

## Data Availability

Raw, anonymized, behavioral data and the analysis scripts for this study are made available on the OSF repository associated with this study (https://osf.io/c3a92/).
