# Supplementary material for: Shared effects of the opioid antagonist naltrexone on first-hand and empathic pain
Source: Soc Cogn Affect Neurosci. 2026 Mar 23;21(1):nsag018. doi: 10.1093/scan/nsag018 (PMC13175174; doi:10.1093/scan/nsag018)
Supplement: nsag018_Supplementary_Data [file nsag018_supplementary_data.docx]

**Supplement to “Shared effects of the opioid antagonist naltrexone on first-hand and empathic pain”**

**1.1 Full list of inclusion/exclusion criteria**

Eligibility criteria included: absence of contraindications for an MRI scan (assessed via an MRI safety-check questionnaire); normal or corrected to normal vision; normal hearing ability; no presence or history of neurological or major medical disorders; right-handedness; regular blood pressure (≤129/84); BMI between 18.5 and 25.0

Exclusion criteria included: known intolerance for naltrexone; current treatment with naltrexone or any other opioidergic agonist/antagonist; consumption of cannabinoids or other consciousness altering drugs 72 hours before the experimental sessions (assessed via urine sample on the day of each experimental session); consumption of alcohol or use of medications 24hours prior to the experimental sessions; pregnancy in female participants (assessed via urine sample on the day of each experimental session)

**1.2 Detailed description including participant instructions and deception**

At each session, the participant and confederate arrived at the same time and were escorted by experimenters to the laboratory, where the consent procedure started. Participants and confederates alike were informed about all relevant study contents, except for the elements of deception (as approved by the local ethics committee). During this, they were told that one person would complete the experiment inside the MRI scanner and receive an MR-signal enhancer (described as improving image quality, similarly to a contrast agent), while the other would perform the task outside the scanner, next to it. Although presented as random, the participant was always assigned to the scanner.

After written consent was obtained, participants and confederates were separated, and the participant completed drug and pregnancy test, followed by a calibration procedure to determine individualized painful and non-painful electrical stimulation intensities (details in supplement, section 2.3). Participants believed that the confederate also underwent this procedure. Afterwards, the electrode remained attached to the participant’s hand.

Next, participants received either naltrexone (Dependex® 50 mg) or placebo (maltodextrin), with order double-blinded and counterbalanced, and emergency unblinding slips were available for severe side effects.

A 45-minute waiting period followed drug administration, during which the confederate re-entered the room and instructions for all experimental paradigms were provided.

For the empathy task in particular, participants and confederates were instructed that they would complete the task together and simultaneously, with the participant in the scanner and the confederate outside, next to it. Both were told that each person’s individually calibrated stimulation intensities would be used during this task.

Participants and confederates were instructed as if the procedure of the task were the same for both: whenever they themselves received a stimulus, a pixelated picture would appear; whenever the other person received a stimulus, a picture of that person’s face would appear. The expression in the pictures corresponded to stimulus intensity: painful expressions for painful stimuli and relaxed expressions for non-painful stimuli. After receiving a stimulus themselves, they were asked to rate how painful it felt to them; after the other person received a stimulus, they were asked to rate how painful they thought the stimulus felt for the other and how unpleasant it was for them to witness.

These pictures were then taken by the experimenters. Both participants and confederates were asked to stand in front of a black screen and perform the expressions. After the photos were taken, they were discarded immediately. All pictures shown in the task were prepared prior to the experiment: confederate photos along with matched pixelated versions were created before the study to ensure consistency in color and luminosity.

After the instructions, most of the 45-minute waiting period had passed. During the remaining time, participants and confederates were offered magazines, but mobile phone use was not allowed. Once the waiting period was complete, participants and confederates were separated again, and the participant completed the Cold Pressor Task (CPT; details reported in the supplement section 2.4).

Next, participants were prepared for the MRI scanner. After they were placed inside, the confederate entered the scanner room. An experimenter escorted them to a table next to the scanner, outside the participant’s view and explained how the confederate would communicate via hand signals as they did not have a microphone. The experimenter then returned to the participant, blocking their view of the door while the confederate quietly left the room. Once this was complete, the experimenter also left the scanner room, and the empathy task began (at > 60 minutes after drug intake).

After the empathy task, another paradigm (not reported here) was completed. When it finished, the confederate re-entered the room while an experimenter again obstructed the participant’s view, so that the confederate’s return was not observed. Both the participant and confederate were then escorted out of the scanner room.

At the end of each session, the participant and confederate were separated to complete questionnaires on a computer. The confederate was made to leave the laboratory shortly after the participant began, giving the impression that both were completing the questionnaires. After the second session, participants completed a brief interview in which the experimenter asked whether they had any doubts about the legitimacy of the study as a whole or specific aspects (e.g. the confederate, the setup of the empathy task, the administered substances, etc.). This was followed by a full debriefing, during which participants were informed about all aspects of deception used in the study.

**2 Additional analyses and details on methods**

**2.1 Psychometric questionnaires**

We administered the following questionnaires after participants had completed both testing sessions:

- Questionnaire of Cognitive and Affective Empathy (QCAE), (Reniers et al., 2011)

- Emotion Contagion Questionnaire (EC), (Doherty, 1997)

- Interpersonal Reactivity Index (IRI), (Davis, 1980)

- Reading the Mind in the Eyes (RM), (Baron-Cohen, Wheelwright, Hill, et al., 2001)

- Empathy Components Questionnaire (ECQ), (Batchelder, Brosnan & Ashwin, 2017)

- Apathy Motivation Index (AMI), (Ang et al., 2017)

- Toronto Alexithymia Scale (TAS), (Bagby et al., 1994)

We examined participants' scores in these questionnaires to identify any outliers that might indicate atypical responses or patterns. The results of the outlier detection can be found in table 1.supp. Since only one extreme outlier (ID 25) was identified on a single questionnaire subscale, no participants were excluded from any analyses based on their questionnaire scores.

| *Table 1.supp. Outlier Table, Questionnaire Subscales* | | | | | |
| --- | --- | --- | --- | --- | --- |
| *Questionnaire* | *Subscale* | *ID* | *Score* | *is.outlier* | *is.extreme* |
| AMI | Emotional Sensitivity | 25 | 3.167 | TRUE | TRUE |
| AMI | Emotional Sensitivity | 34 | 2.333 | TRUE | FALSE |
| AMI | Emotional Sensitivity | 35 | 2.333 | TRUE | FALSE |
| EC | Love | 9 | 8.000 | TRUE | FALSE |
| EC | Love | 18 | 8.000 | TRUE | FALSE |
| EC | Love | 34 | 6.000 | TRUE | FALSE |
| ECQ | Affective Ability | 18 | 8.000 | TRUE | FALSE |
| ECQ | Cognitive Ability | 10 | 12.000 | TRUE | FALSE |
| ECQ | Cognitive Drive | 18 | 10.000 | TRUE | FALSE |
| QCAE | Perspective Taking | 2 | 19.000 | TRUE | FALSE |
| TAS | Identifying Feelings | 15 | 29.000 | TRUE | FALSE |

Note: AMI = Apathy Motivation Index, EC = Emotional Contagion Scale, ECQ = Empathy Components Questionnaire, QCAE = Questionnaire of Cognitive and Affective Empathy, TAS = Toronto Alexithymia Scale

**2.2 *Post-experimental ratings, confederates.***

Each testing session participants arrived together with a female confederate of the experimenters, posing as another participant (the same confederate each session). In total there were seven different confederates. All confederates were rated similarly across rating conditions and there was no difference in average ratings between the two drugs (see table 2.supp).

| *Table 2.supp. No Differences in Participant’s overall Judgement of the Confederates between Drugs* | | | | | | | | |
| --- | --- | --- | --- | --- | --- | --- | --- | --- |
|  | Placebo | | Naltrexone | |  | | | |
|  | *M* | *SD* | *M* | *SD* | *df* | *t* | *p* | *Cohen’s d* |
| Similarity | 5.829 | 2.189 | 6.000 | 1.627 | 34 | -0.518 | 0.609 | -0.09 |
| Weakness | 5.971 | 1.361 | 5.914 | 1.541 | 34 | 0.201 | 0.842 | 0.03 |
| Closeness | 4.486 | 1.755 | 4.371 | 1.816 | 34 | 0.327 | 0.746 | 0.06 |
| Liking | 3.029 | 1.505 | 3.057 | 1.748 | 34 | -0.167 | 0.869 | -0.03 |
| Agreeableness | 3.000 | 1.283 | 2.743 | 1.400 | 34 | 1.246 | 0.221 | 0.21 |
| Neediness | 6.543 | 1.771 | 6.171 | 2.189 | 34 | 0.982 | 0.333 | 0.17 |

Note: M = Mean, SD = Standard Deviation. Ratings on the other person have been collected via 5-point-Likert scales with contrasting characteristics on each end of the scale (e.g. dissimilar – similar, weak – strong, etc.).

**2.3 Calibration of electrical pain thresholds**

Participants’ pain threshold was calibrated using the DS5 Isolated Bipolar Constant Current Stimulator (Digitimer Ltd.). A series of short electrical stimuli (500ms) were administered via a concentric electrode, fixated on participants’ left hand (dorsum manus, between thumb and index finger). After each stimulus participants rated it on a seven-point Likert scale (1 = perceptible but not painful, 7 = extremely painful). For the Empathy for Pain Paradigm the stimuli most consistently rated as 1 were used for the non-painful stimuli in the task, while those rated as 6 were used for the painful stimuli. Participants were kept blind regarding the exact stimulus intensities administered. When comparing the average calibrated stimulus intensities per drug, we did not detect a significant difference (see table 3.supp).

| *Table 3.supp. No Differences in Participant’s Average Calibrated Electrical Stimulus Intensities between Drugs* | | | | | | | | |
| --- | --- | --- | --- | --- | --- | --- | --- | --- |
|  | Placebo | | Naltrexone | |  | | | |
|  | *M* | *SD* | *M* | *SD* | *df* | *t* | *p* | *Cohen’s d* |
| Painful Stimuli | 0.791 | 0.120 | 0.987 | 0.307 | 34 | -0.89 | 0.382 | -0.15 |
| Non-Painful Stimuli | 0.253 | 0.058 | 0.237 | 0.039 | 34 | -0.44 | 0.663 | -0.07 |

Note: M = Mean, SD = Standard Deviation. Two-tailed paired t-tests were conducted. Calibrated electrical stimulus intensities given in mA.

**2.4 Cold Pressor Task details**

We filled a container with 3 liters of water, cooled with ice packs. The temperature was monitored with a lab thermometer (LT-101, TFA Dostmann GmbH & Co. KG), and we kept the water circulated using a CompactON 300 pump (EHEIM GmbH & Co. KG). Water temperature ranged from 1.1 – 5.5 °C. There was no significant difference in water temperature for the two drug [paired t-test, t(34) = 1.43, *p* = 0.163, two-tailed, Cohen’s d = 0.24].

*Naltrexone*: mean temp. ± S.E.M = 2.686°C ± 0.121°C

*Placebo:* mean temp. ± S.E.M = 2.809°C ± 0.150°C


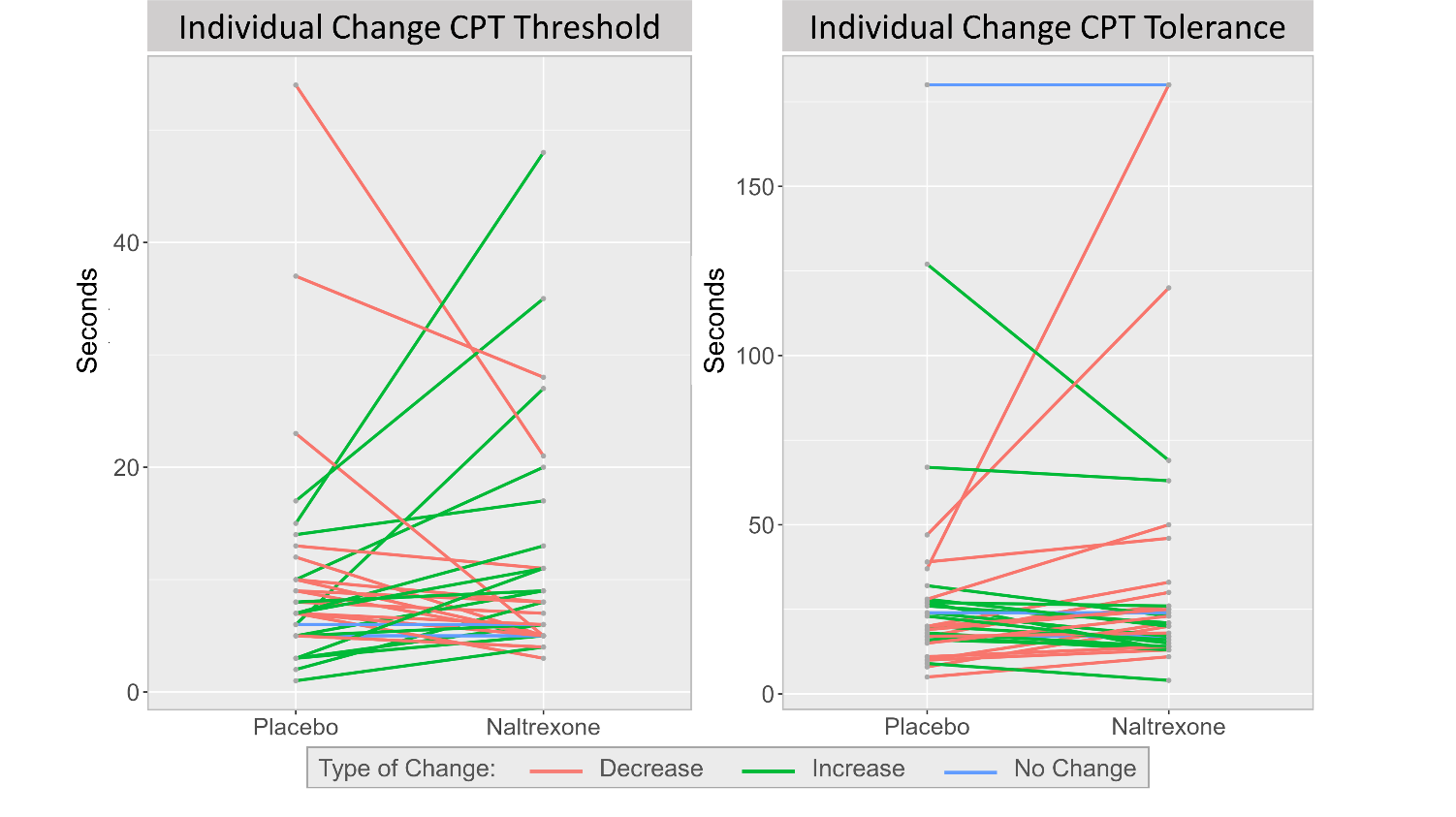


**Figure 1.supp** Cold Pressor Task (CPT) data (n = 35). Left: individual change in CPT threshold (how long it took until participants experienced pain while having their hand submerged in cold water, measured in seconds) between the two drugs. Right: individual change in CPT tolerance (how long participants were able to keep their hands submerged in the cold water before they had to remove it, measured in seconds) between the two drugs. The different line colors represent the types of change in individual CPT Threshold and Tolerance induced via administration of Naltrexone as compared to Placebo.

To complement the frequentist analysis approach CPT data was also analyzed using Bayesian statistics. A Bayesian Wilcoxon signed rank test (*dfba_wilcoxon;* Barch & Chechile, 2023) comparing the CPT threshold between the placebo and naltrexone sessions revealed moderate evidence in favor of the null hypothesis, with a Bayes factor (BF_10_) of 0.411 (±0.01%). Similarly, for the CPT tolerance the Bayesian Wilcoxon signed rank test yielded a Bayes factor of 0.202 (±0.01%), indicating moderate evidence in favor of the null hypothesis as well.

While peak plasma concentrations of orally administered naltrexone are typically reached within 1 hour (Gonzalez & Brogden, 1988; Paderni et al., 2013), evidence linking this peak with mu-opioid receptor blockade is lacking (Trøstheim et al., 2023). Given the likely temporal relationship between plasma concentration and receptor blockade, it is reasonable to assume that effective blockade requires at least 1 hour. This difference in timing could account for the absence of significant effects in the CPT compared to the Empathy for Pain task, which occurred 1 hour after substance administration and showed measurable effects. Following the lead of Trøstheim and colleagues (2023) for future studies we thus recommend waiting at minimum one hour before any psychological assessments.

**2.5 Empathy for Pain Task details**

| *Table 4.supp Results Table for rmANOVAs on Self-Report Ratings in the Empathy for Pain Task* | | | | | | | |
| --- | --- | --- | --- | --- | --- | --- | --- |
| *Pain Ratings* | | | | | | | |
|  | *df_Num_* | *df_Den_* | *MSE* | *F* | *p* | *η_p_^2^* |  |
| Drug | 1 | 34 | 0.656 | 1.474 | 0.233 | 0.042 |  |
| Recipient | 1 | 34 | 0.918 | 0.406 | 0.528 | 0.012 |  |
| Intensity*** | 1 | 34 | 1.575 | 699.516 | < 0.001 | 0.954 |  |
| Drug x Recipient | 1 | 34 | 0.364 | 0.303 | 0.586 | 0.009 |  |
| Drug x Intensity* | 1 | 34 | 0.512 | 7.444 | 0.010 | 0.180 |  |
| Recipient x Intensity*** | 1 | 34 | 0.551 | 24.116 | < 0.001 | 0.415 |  |
| Drug x Recipient x Intensity | 1 | 34 | 0.445 | 3.328 | 0.077 | 0.089 |  |
| *Unpleasantness Ratings* |  |  |  |  |  |  |  |
| Drug | 1 | 34 | 0.401 | 0.521 | 0.475 | 0.015 |  |
| Intensity*** | 1 | 34 | 0.821 | 363.206 | < 0.001 | 0.914 |  |
| Drug x Intensity* | 1 | 34 | 0.247 | 4.432 | 0.043 | 0.115 |  |

Note: df_Num_ = degrees of freedom numerator, df_Den_ = degrees of freedom denominator, MSE = mean squared error, η_p_^2^ = partial eta-sqaured. Results are reported using Greenhouse Geisser sphericity correction.

**Simple effects and post-hoc comparisons, cognitive-evaluative aspect of empathy rmANOVA (Bonferroni-adjusted):**

Simple effects analyses revealed no significant main effect of drug (Placebo vs. Naltrexone: *estimate* = 0.118, *SE* = 0.097, *t*(34) = 1.21, *p* = .233) and no significant difference between self- and other-directed ratings (*estimate* = -0.073, *SE* = 0.114, *t*(34) = -0.64, *p* = .528). Painful stimuli were rated significantly more painful than non-painful stimuli (*estimate* = 3.97, *SE* = 0.15, *t*(34) = 26.45, *p* < .0001).

Post-hoc pairwise comparisons of drug within each intensity level indicated that naltrexone selectively reduced ratings for painful stimuli (*estimate* = 0.351, *SE* = 0.132, *t*(34) = 2.65, *p* = .012; emmeans: Placebo = 5.74, Naltrexone = 5.39), whereas ratings for non-painful stimuli were unaffected (*estimate* = -0.116, *SE* = 0.126, *t*(34) = -0.92, *p* = .364; emmeans: Placebo = 1.54, Naltrexone = 1.66).

Post-hoc pairwise comparisons of recipient within each intensity level revealed that for painful stimuli, self-directed ratings were higher than other-directed ratings (estimate = 0.363, SE = 0.149, t(34) = 2.43, *p* = 0.021; emmeans: Self = 5.75, Other = 5.39). For non-painful stimuli, other-directed ratings were higher than self-directed ratings (estimate = -0.509, SE = 0.140, t(34) = -3.63, *p* = 0.001; emmeans: Self = 1.35, Other = 1.85).

**Simple effects and post-hoc comparisons, affective-motivational aspect of empathy rmANOVA (Bonferroni-adjusted):**

Simple effects analyses revealed no significant main effect of drug (Placebo vs. Naltrexone: *estimate* = -0.0773, *SE* = 0.107, *t*(34) = -0.722, *p* = 0.475). However, painful stimuli were rated significantly more painful than non-painful stimuli (*estimate* = 2.92, *SE =* 0.153, *t*(34) = 19.06, *p* < .0001).

Post-hoc pairwise comparisons of drug within each intensity level showed no significant difference in unpleasantness ratings between drug in either painful (estimate = 0.100, SE = 0.122, t(34) = 0.82, *p* = 0.42; emmeans: Placebo = 4.63, Naltrexone = 4.53) or non-painful stimuli (estimate = -0.254, SE = 0.149, t(34) = -1.71, *p* = 0.097; emmeans: Placebo = 1.53, Naltrexone = 1.79).


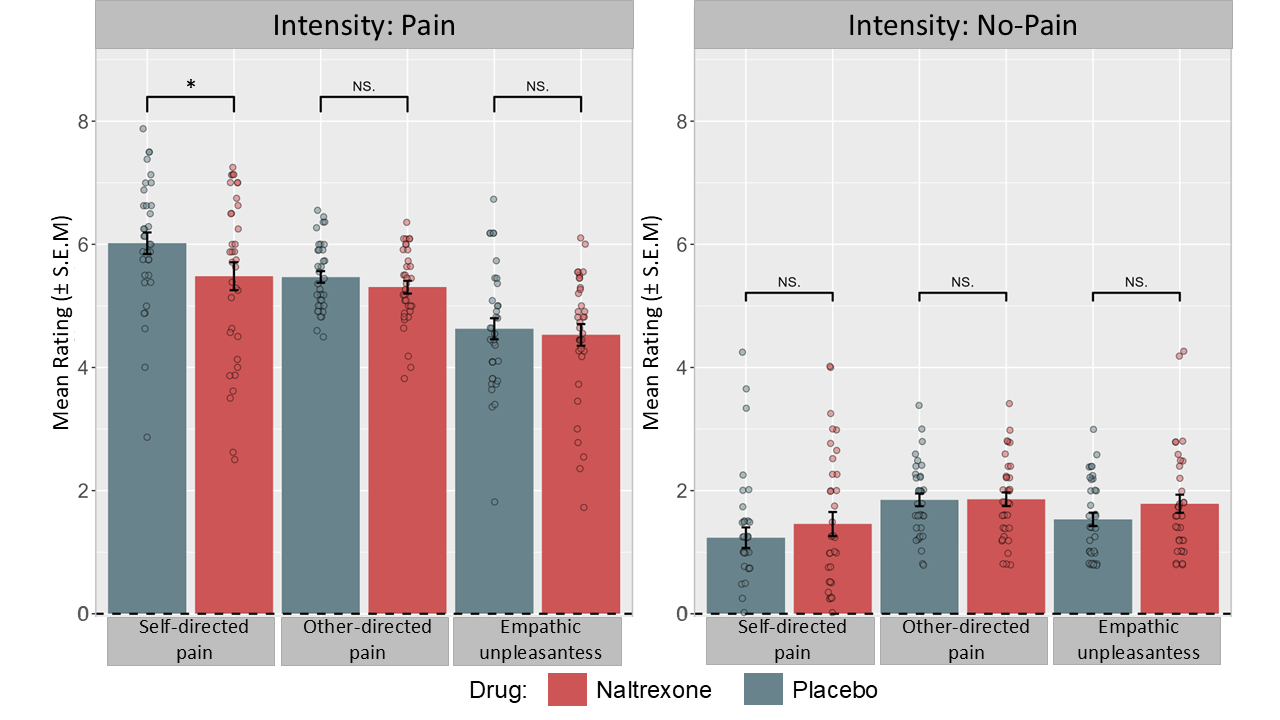


**Figure 2.supp** Empathy for Pain Task self-report data (n=35), Mean ratings for stimuli administered either to oneself (self-directed pain: “How painful was this stimulus for you?” or to another person (other-directed pain: “How painful was this stimulus for the other person?”; empathic unpleasantness: “How unpleasant did it feel, when the other person was stimulated?”). Ratings were given on a 9-point Likert scale. Separate plots for painful stimuli (left) vs. non-painful stimuli (right).

| *Table 5.supp Results Table for Bayesian ANOVAs on Self-Report Ratings in the Empathy for Pain Task* | | |
| --- | --- | --- |
| *Pain Ratings* | | |
|  | BF_10_ | Posterior Probability |
| Drug | 0.142 | < 0.001 |
| Recipient | 0.134 | < 0.001 |
| Intensity | 3.424123e+106 | 0.004 |
| Drug x Recipient | 0.004 | < 0.001 |
| Drug x Intensity | 1.457737e+106 | 0.002 |
| Recipient x Intensity | 4.251730e+108 | 0.503 |
| Drug x Recipient x Intensity | 2.438478e+107 | 0.029 |
| Unpleasantness Ratings |  |  |
| Drug | 0.182 | < 0.001 |
| Intensity | 8.383660e+44 | 0.739 |
| Drug x Intensity | 1.183210e+44 | 0.104 |

Again, complementary to the frequentist analysis of the Empathy for Pain data, Bayesian statistics were conducted using the function anovaBF from the R package “BayesFactor” (Morey & Rouder, 2024).

The Bayesian analysis aligned with the frequentist results, showing no evidence for a main effect of drug or its interactions, while strongly supporting the effects of pain intensity.

Additionally, Bayesian one-sample t-tests were conducted for the comparisons of self-directed pain, other-directed pain, and other-directed unpleasantness between the naltrexone and placebo sessions (self-directed pain: BF₁₀ = 3.33, other-directed pain: BF₁₀ = 0.47, other-directed unpleasantness: BF₁₀ = 1.54).

**3 Exploratory analyses**

**3.1 Correlation between all Task Change Indices**

| *Table 6.supp Descriptive Statistics and Correlations for Indices of Difference in Study Variables between the two Drugs* | | | | | | | | | |
| --- | --- | --- | --- | --- | --- | --- | --- | --- | --- |
| *Contrast* | | | | | | | | | |
|  | *n* | *M* | *SD* | *1* | *2* | *3* | *4* | *5* |  |
| 1 CPT Threshold (NAL-PLA) | 35 | 0.886 | 10.501 | _ |  |  |  |  |  |
| 2 CPT Tolerance (NAL-PLA) | 35 | 5.800 | 29.661 | 0.53 | _ |  |  |  |  |
| 3 EMP Self-directed Pain Ratings  (Pain-No Pain; NAL-PLA) | 35 | -0.176 | 0.844 | -0.06 | -0.04 | _ |  |  |  |
| 4 EMP Other-directed Pain Ratings  (Pain-No Pain; NAL-PLA) | 35 | -0.354 | 0.995 | 0.02 | 0.04 | -0.22 | _ |  |  |
| 5 EMP Empathic Unpleasantness Ratings  (Pain-No Pain; NAL-PLA) | 35 | -0.757 | 1.765 | -0.15 | -0.22 | 0.24 | 0.68 | _ |  |

Note: M = Mean, SD = Standard Deviation.

**3.2 Comparison of naltrexone effects on firsthand pain and empathic unpleasantness.**

To assess if the effects of naltrexone on firsthand pain and unpleasantness regarding another person’s pain were of similar magnitude, we explored whether there was an interaction between drug and rating type (self-directed pain vs. empathic unpleasantness). To do so, we conducted a rmANOVA on those two ratings and included rating type and drug as within-subject factors. While there was a main effect for rating type [*F*(1,34) = 9.426, *p* = 0.004, *η_p_^2^* = 0. 0.217], the interaction between those factors was not significant [*F*(1,34) = 3.115, *p* =0.087 , *η_p_^2^* = 0.084]. This indicates that the effects of naltrexone on participants’ firsthand pain and their affective reaction towards another person’s pain were not statistically different.

| *Table 7.supp Results Table for exploratory rmANOVA* | | | | | | | |
| --- | --- | --- | --- | --- | --- | --- | --- |
|  | *df_Num_* | *df_Den_* | *MSE* | *F* | *p* | *η_p_^2^* |  |
| Drug | 1 | 34 | 0.450 | 0.124 | 0.726 | 0.004 |  |
| Rating Type** | 1 | 34 | 0.682 | 9.426 | 0.004 | 0.217 |  |
| Drug x Rating Type | 1 | 34 | 0.155 | 3.115 | 0.087 | 0.084 |  |

Note: df_Num_ = degrees of freedom numerator, df_Den_ = degrees of freedom denominator, MSE = mean squared error, η_p_^2^ = partial eta-sqaured. Results are reported using Greenhouse Geisser sphericity correction.

| *Table 8.supp Results Table for Bayesian exploratory rmANOVA* | | |
| --- | --- | --- |
|  | BF_10_ | Posterior Probability |
| Drug | 0.130 | 0.183 |
| Rating Type | 0.502 | 0.707 |
| Drug x Rating Type | 0.013 | 0.018 |

The Bayesian exploratory analysis provided strong evidence against an interaction between drug and rating type, as indicated by a low Bayes factor (BF₁₀ = 0.013) and a posterior probability of only 0.018. This suggests that drug did not differentially affect the two rating types.

**3.3 Sex differences**

As some studies reported sex-dependent differences in the effect of naltrexone on experimental pain (al’Absi et al., 2004; France et al., 2005) we were interested, whether we could find similar effects in our data. Thus, we explored whether naltrexone differentially modulated the responses in female vs. male participants towards their own pain, as well as their empathy for another person’s pain. Generally, sex differences in pain sensitivity have been investigated manifold and the picture that emerges suggests, that overall women may have higher pain sensitivity, as compared to men (Casale et al., 2021; Mogil, 2012). Moreover, both sexes also differ in their empathy, with women reporting greater affective responsiveness as compared to men (Christov-Moore et al., 2014). To detect potential interactions of naltrexone with sex on ratings of firsthand pain and empathy for pain, we conducted two mixed ANOVAs (separately for pain ratings, as well as unpleasantness ratings) including the between-subjects factor sex (female vs. male) and the within-subjects factors drug (naltrexone vs. placebo), intensity (pain vs. no-pain) and for the pain ratings mixed ANOVA the additional within-subjects factor recipient (self vs. other). The overall pattern of results remained unchanged, and no significant interactions involving sex and drug were observed (see table 9.supp).

| *Table 9.supp Results Table for rmANOVAs on Self-Report Ratings in the Empathy for Pain Task, including the Between-Subjects Factor Sex (Female vs. Male)* | | | | | | | |
| --- | --- | --- | --- | --- | --- | --- | --- |
|  | *df_Num_* | *df_Den_* | *MSE* | *F* | *p* | *η_p_^2^* |  |
| *Pain Ratings* |  |  |  |  |  |  |  |
| Sex | 1 | 33 | 1.718 | 0.032 | 0.858 | 0.001 |  |
| Drug | 1 | 33 | 0.660 | 1.860 | 0.182 | 0.053 |  |
| Sex x Drug | 1 | 33 | 0.660 | 0.789 | 0.381 | 0.023 |  |
| Recipient | 1 | 33 | 0.915 | 0.698 | 0.409 | 0.021 |  |
| Sex x Recipient | 1 | 33 | 0.915 | 1.102 | 0.301 | 0.032 |  |
| Intensity*** | 1 | 33 | 1.271 | 867.694 | < 0.001 | 0.963 |  |
| Sex x Intensity** | 1 | 33 | 1.271 | 9.146 | 0.005 | 0.217 |  |
| Drug x Recipient | 1 | 33 | 0.372 | 0.410 | 0.527 | 0.012 |  |
| Sex x Drug x Recipient | 1 | 33 | 0.372 | 0.282 | 0.599 | 0.008 |  |
| Drug x Intensity* | 1 | 33 | 0.509 | 6.065 | 0.019 | 0.155 |  |
| Sex x Drug x Intensity | 1 | 33 | 0.509 | 1.184 | 0.284 | 0.035 |  |
| Recipient x Intensity*** | 1 | 33 | 0.557 | 24.359 | < 0.001 | 0.425 |  |
| Sex x Recipient x Intensity | 1 | 33 | 0.557 | 0.587 | 0.449 | 0.017 |  |
| Drug x Recipient x Intensity | 1 | 33 | 0.458 | 3.170 | 0.084 | 0.088 |  |
| Sex x Drug x Recipient x Intensity | 1 | 33 | 0.458 | 0.009 | 0.924 | 0.000 |  |
| *Unpleasantness Ratings* |  |  |  |  |  |  |  |
| Sex | 1 | 33 | 1.839 | 0.303 | 0.585 | 0.009 |  |
| Drug | 1 | 33 | 0.410 | 0.351 | 0.558 | 0.011 |  |
| Sex x Drug | 1 | 33 | 0.410 | 0.289 | 0.595 | 0.009 |  |
| Intensity*** | 1 | 33 | 0.607 | 503.769 | < 0.001 | 0.939 |  |
| Sex x Intensity** | 1 | 33 | 0.607 | 13.016 | 0.001 | 0.283 |  |
| Drug x Intensity | 1 | 33 | 0.231 | 3.119 | 0.087 | 0.086 |  |
| Sex x Drug x Intensity | 1 | 33 | 0.231 | 3.383 | 0.075 | 0.093 |  |

Note: df_Num_ = degrees of freedom numerator, df_Den_ = degrees of freedom denominator, MSE = mean squared error, η_p_^2^ = partial eta-sqaured. Results are reported using Greenhouse Geisser sphericity correction.

**3.4 Blood Pressure**

As administration of naltrexone may increase blood pressure (O’brien et al., 1975), we measured it following the waiting period using an upper arm blood pressure monitor. We then compared both systolic and diastolic blood pressure between the naltrexone and the placebo session using two dependent samples t-tests (see table 10.supp for results). We had to exclude two participants for these analyses, as their blood pressure data for the naltrexone session was not recorded.

| *Table 10.supp No Differences in Participant’s Average Systolic and Diastolic Blood Pressure (BP) between Drugs* | | | | | | | | |
| --- | --- | --- | --- | --- | --- | --- | --- | --- |
|  | Placebo | | Naltrexone | |  | | | |
|  | *M* | *SD* | *M* | *SD* | *df* | *t* | *p* | *Cohen’s d* |
| Systolic BP | 125.939 | 13.544 | 124.758 | 13.673 | 34 | -0.55 | 0.587 | -0.10 |
| Diastolic BP | 82.545 | 8.938 | 80.939 | 8.707 | 34 | -0.88 | 0.383 | -0.15 |

Note: M = Mean, SD = Standard Deviation. Two-tailed paired t-tests were conducted. N = 33

Moreover, since in some studies an impact of blood pressure on the effect of naltrexone on experimental pain was reported (Kotlyar et al., 2008; McCubbin & Bruehl, 1994), we conducted an exploratory analysis to examine whether we could replicate such findings. This analysis included two Linear Mixed Models (LMM) using the *lme* function of the R package “lme4” (Bates et al., 2015) each for the effects of systolic or diastolic blood pressure on either pain ratings or unpleasantness ratings within the Empathy for Pain Task. Apart from the significant intercepts in the diastolic blood pressure models, no predictors reached significance, indicating that blood pressure did not influence the relationship between either pain or unpleasantness ratings in our study.

| *Table 11.supp LMM results for the effects of systolic blood pressure on pain ratings overall.* | | | | | |
| --- | --- | --- | --- | --- | --- |
| **Pain ratings Systolic BP Model** | | | | | |
| *Fixed Effects* | | | | | |
|  | *Estimate* | *SE* | *t* | *p* | *95% CI* |
| (Intercept) | 3.62 | 1.90 | 1.90 | 0.058 | -0.13 – 7.37 |
| Drug (Placebo) | -0.49 | 2.67 | -0.19 | 0.853 | -5.76 – 4.77 |
| Blood Pressure | -0.00 | 0.002 | -0.03 | 0.973 | -0.03 – 0.03 |
| Drug (Placebo) x Blood Pressure | 0.00 | 0.02 | 0.23 | 0.821 | -0.04 – 0.05 |
|  | | | | | |
| *Random Effects* | | | | | |
|  | *Variance* | *SD* | *Correlation* | | |
| Participant (Intercept) | 5.671 | 2.381 |  |  |  |
| Drug (Placebo) | 3.891 | 1.972 | -0.03 |  |  |
| Blood Pressure | 0.000 | 0.019 | -1.00 | 0.02 |  |
| Drug (Placebo) x Blood Pressure | 0.000 | 0.015 | 0.04 | -1.00 | -0.03 |
|  |  |  |  |  |  |
| *Model fit* | | | | | |
| R² | Marginal | Conditional |  |  |  |
|  | 0.001 | - |  |  |  |

Note: SE = standard error, SD = standard deviation. p-values for fixed effects calculated using Satterthwaites approximations. All p-values are based on two-sided tests (uncorrected). Confidence Intervals (CI) have been calculated using the Wald method. Model equation: Pain Rating ~ Drug*Blood Pressure + (1+Drug*Blood Pressure|Participant).

| *Table 12.supp Linear mixed model results for the effects of diastolic blood pressure on pain ratings overall.* | | | | | |
| --- | --- | --- | --- | --- | --- |
| **Pain ratings Diastolic BP Model** | | | | | |
| *Fixed Effects* | | | | | |
|  | *Estimate* | *SE* | *t* | *p* | *95% CI* |
| (Intercept)* | 4.04 | 1.94 | 2.08 | 0.038 | 0.22 – 7.68 |
| Drug (Placebo) | -1.73 | 2.71 | -0.64 | 0.523 | -7.07 – 3.60 |
| Blood Pressure | -0.01 | 0.02 | -0.25 | 0.804 | -0.05 – 0.04 |
| Drug (Placebo) x Blood Pressure | 0.02 | 0.03 | 0.68 | 0.497 | -0.04 – 0.09 |
|  | | | | | |
| *Random Effects* | | | | | |
|  | *Variance* | *SD* | *Correlation* | | |
| Participant (Intercept) | 3.745 | 1.935 |  |  |  |
| Drug (Placebo) | 4.810 | 2.193 | -0.22 |  |  |
| Blood Pressure | 0.000 | 0.024 | -1.00 | 0.22 |  |
| Drug (Placebo) x Blood Pressure | 0.000 | 0.027 | 0.23 | -1.00 | -0.23 |
|  |  |  |  |  |  |
| *Model fit* | | | | | |
| R² | Marginal | Conditional |  |  |  |
|  | 0.003 | - |  |  |  |

Note: SE = standard error, SD = standard deviation. p-values for fixed effects calculated using Satterthwaites approximations. All p-values are based on two-sided tests (uncorrected). Confidence Intervals (CI) have been calculated using the Wald method. Model equation: Pain Rating ~ Drug*Blood Pressure + (1+Drug*Blood Pressure|Participant).

| *Table 13.supp Linear mixed model results for the effects of systolic blood pressure on unpleasantness ratings overall.* | | | | | |
| --- | --- | --- | --- | --- | --- |
| **Unpleasantness ratings Systolic BP Model** | | | | | |
| *Fixed Effects* | | | | | |
|  | *Estimate* | *SE* | *t* | *p* | *95% CI* |
| (Intercept) | 3.35 | 2.01 | 1.67 | 0.098 | -0.63 – 7.33 |
| Drug (Placebo) | -0.84 | 2.87 | -0.29 | 0.770 | -6.53 – 4.84 |
| Blood Pressure | -0.00 | 0.02 | -0.08 | 0.939 | -0.03 – 0.03 |
| Drug (Placebo) x Blood Pressure | 0.01 | 0.02 | 0.26 | 0.797 | -0.04 – 0.05 |
|  | | | | | |
| *Random Effects* | | | | | |
|  | *Variance* | *SD* | *Correlation* | | |
| Participant (Intercept) | 0.000 | 0.000 |  |  |  |
|  |  |  |  |  |  |
| *Model fit* | | | | | |
| R² | Marginal | Conditional |  |  |  |
|  | 0.002 | - |  |  |  |

Note: SE = standard error, SD = standard deviation. p-values for fixed effects calculated using Satterthwaites approximations. All p-values are based on two-sided tests (uncorrected). Confidence Intervals (CI) have been calculated using the Wald method. Model equation: Unpleasantness Rating ~ Drug*Blood Pressure + (1|Participant).

| *Table 14.supp Linear mixed model results for the effects of diastolic blood pressure on unpleasantness ratings overall.* | | | | | |
| --- | --- | --- | --- | --- | --- |
| **Unpleasantness ratings Diastolic BP Model** | | | | | |
| *Fixed Effects* | | | | | |
|  | *Estimate* | *SE* | *t* | *p* | *95% CI* |
| (Intercept)* | 4.07 | 2.05 | 1.99 | 0.049 | 0.02 – 8.12 |
| Drug (Placebo) | -2.03 | 2.88 | -0.70 | 0.483 | -7.74 – 3.68 |
| Blood Pressure | -0.01 | 0.03 | -0.43 | 0.669 | -0.06 – 0.04 |
| Drug (Placebo) x Blood Pressure | 0.02 | 0.04 | 0.67 | 0.503 | -0.05 – 0.09 |
|  | | | | | |
| *Random Effects* | | | | | |
|  | *Variance* | *SD* | *Correlation* | | |
| Participant (Intercept) | 0.000 | 0.000 |  |  |  |
|  |  |  |  |  |  |
| *Model fit* | | | | | |
| R² | Marginal | Conditional |  |  |  |
|  | 0.004 | - |  |  |  |

Note: SE = standard error, SD = standard deviation. p-values for fixed effects calculated using Satterthwaites approximations. All p-values are based on two-sided tests (uncorrected). Confidence Intervals (CI) have been calculated using the Wald method. Model equation: Unpleasantness Rating ~ Drug*Blood Pressure + (1|Participant).

**3.5 Details for exploratory mediation analyses**


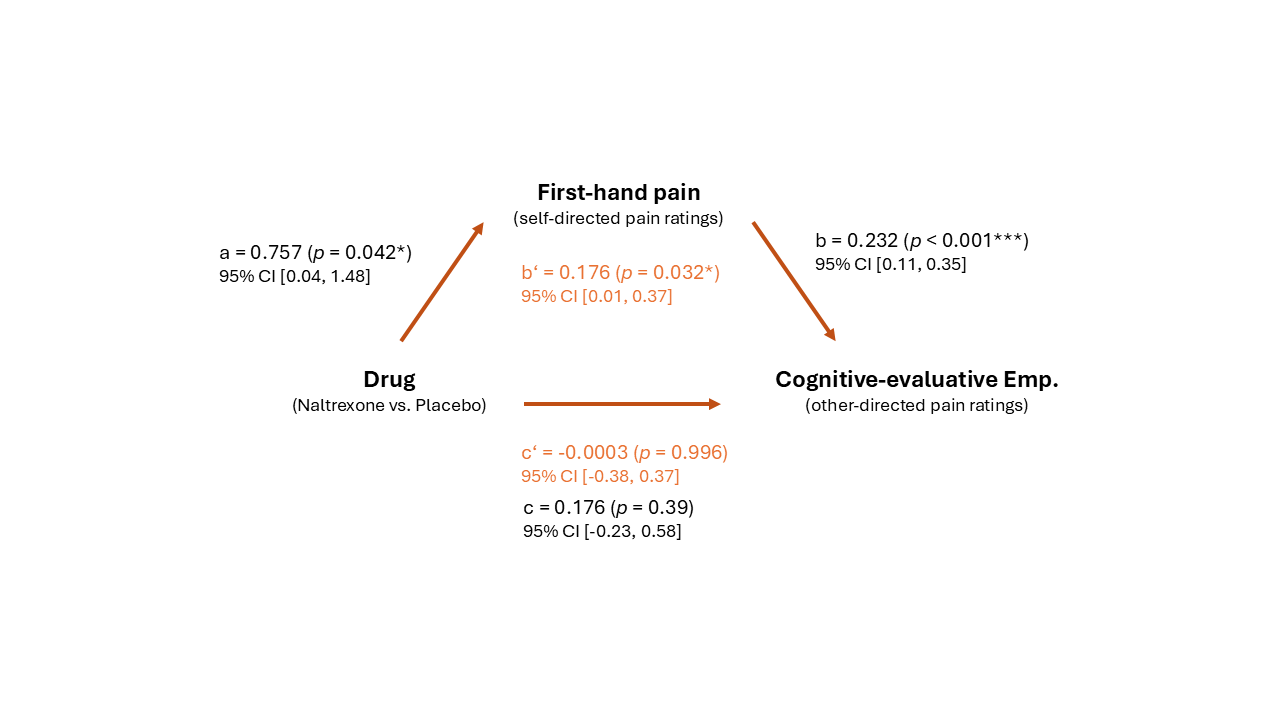


Figure 3.supp: Mediation model 1 – cognitive-evaluative empathy


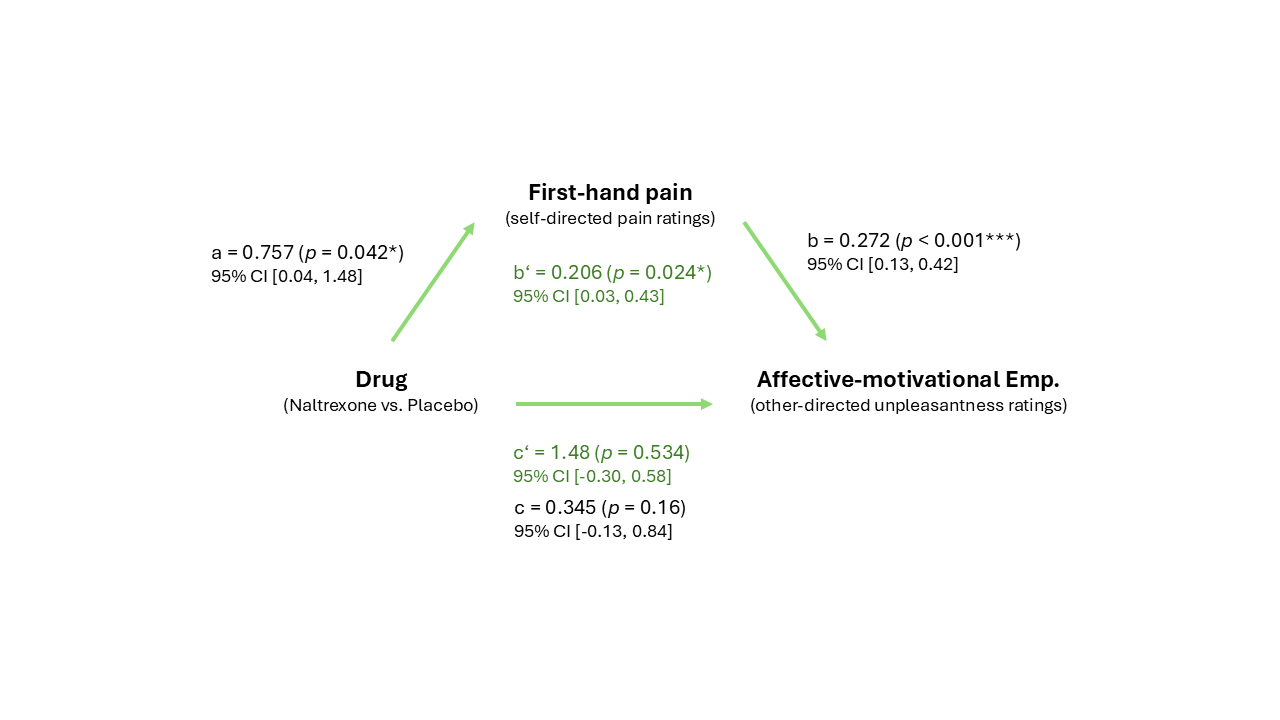


Figure 4.supp: Mediation model 2 – affective-motivational empathy

**4. References for Supplement**

al’Absi, M., Wittmers, L. E., Ellestad, D., Nordehn, G., Kim, S. W., Kirschbaum, C., & Grant, J. E. (2004). Sex Differences in Pain and Hypothalamic-Pituitary-Adrenocortical Responses to Opioid Blockade. *Psychosomatic Medicine*, *66*(2).

Barch, D., & Chechile, R. (2023). *DFBA: Distribution-Free Bayesian Analysis* (Version R package version 0.1.0) [R]. <https://CRAN.R-project.org/package=DFBA>

Bates, D., Maechler, M., Bolker, B., & Walker, S. (2015). Fitting Linear Mixed-Effects Models Using lme4. *Journal of Statistical Software*, *67*(1), 1–48. https://doi.org/doi:10.18637/jss.v067.i01

Casale, R., Atzeni, F., Bazzichi, L., Beretta, G., Costantini, E., Sacerdote, P., & Tassorelli, C. (2021). Pain in Women: A Perspective Review on a Relevant Clinical Issue that Deserves Prioritization. *Pain and Therapy*, *10*(1), 287–314. https://doi.org/10.1007/s40122-021-00244-1

Christov-Moore, L., Simpson, E. A., Coudé, G., Grigaityte, K., Iacoboni, M., & Ferrari, P. F. (2014). Empathy: Gender effects in brain and behavior. *Neuroscience & Biobehavioral Reviews*, *46*, 604–627. https://doi.org/10.1016/j.neubiorev.2014.09.001

France, C. R., al’Absi, M., Ring, C., France, J. L., Brose, J., Spaeth, D., Harju, A., Nordehn, G., & Wittmers, L. E. (2005). Assessment of opiate modulation of pain and nociceptive responding in young adults with a parental history of hypertension. *Biological Psychology*, *70*(3), 168–174. https://doi.org/10.1016/j.biopsycho.2005.01.012

Gonzalez, J. P., & Brogden, R. N. (1988). Naltrexone. *Drugs*, *35*(3), 192–213. https://doi.org/10.2165/00003495-198835030-00002

Kotlyar, M., al’Absi, M., Brauer, L. H., Grant, J. E., Fong, E., & Kim, S. W. (2008). Naltrexone effect on physiological and subjective response to a cold pressor task. *Biological Psychology*, *77*(2), 233–236. https://doi.org/10.1016/j.biopsycho.2007.10.005

McCubbin, J. A., & Bruehl, S. (1994). Do endogenous opioids mediate the relationship between blood pressure and pain sensitivity in normotensives? *Pain*, *57*(1), 63–67. https://doi.org/10.1016/0304-3959(94)90108-2

Mogil, J. S. (2012). Sex differences in pain and pain inhibition: Multiple explanations of a controversial phenomenon. *Nature Reviews Neuroscience*, *13*(12), 859–866. https://doi.org/10.1038/nrn3360

Morey, R., & Rouder, J. (2024). *_BayesFactor: Computation of Bayes Factors for Common Designs_* (Version 0.9.12-4.7) [Computer software]. https://CRAN.R-project.org/package=BayesFactor

O’brien, C. P., Greenstein, R. A., Mintz, J., & Woody, G. E. (1975). Clinical Experience with Naltrexone. *The American Journal of Drug and Alcohol Abuse*, *2*(3–4), 365–377. https://doi.org/10.3109/00952997509005662

Paderni, C., Campisi, G., Schumacher, A., Göttsche, T., Giannola, L. I., De Caro, V., & Wolff, A. (2013). Controlled delivery of naltrexone by an intraoral device: In vivo study on human subjects. *International Journal of Pharmaceutics*, *452*(1), 128–134. https://doi.org/10.1016/j.ijpharm.2013.04.070

Trøstheim, M., Eikemo, M., Haaker, J., Frost, J. J., & Leknes, S. (2023). Opioid antagonism in humans: A primer on optimal dose and timing for central mu-opioid receptor blockade. *Neuropsychopharmacology*, *48*(2), 299–307. https://doi.org/10.1038/s41386-022-01416-z
